# Impact of gestational weight gain on perineal injury in overweight and obese primiparous women

**DOI:** 10.1007/s00404-025-08291-1

**Published:** 2026-01-05

**Authors:** Itamar Gilboa, Daniel Gabbai, Emmanuel Attali, Ronen Gold, Asnat Groutz, Yariv Yogev, Yoav Baruch

**Affiliations:** 1 Department of Obstetrics and Gynecology, Lis Hospital for Women’s Health, Tel Aviv Sourasky Medical Center, 6 Weizmann St., 6423906 Tel Aviv, Israel; 2 Gray Faculty of Medicine, Tel Aviv University, Tel Aviv, Israel

**Keywords:** Overweight, Obesity, Body mass index (BMI), High BMI, Gestational weight gain (GWG), Perineal injury, Perineal tears, Obstetric anal sphincter injury (OASI)

## Abstract

**Objective:**

Overweight and obesity are associated with adverse pregnancy outcomes, yet higher body mass index (BMI) has paradoxically been linked to reduced risk of perineal lacerations, including obstetric anal sphincter injury (OASI). The role of gestational weight gain (GWG) in this context is not well established. The objective of this study was to evaluate the association between GWG and perineal injury among overweight and obese primiparous women.

**Methods:**

A retrospective cohort study was conducted at a tertiary center between 2012–2023, including primiparous women with singleton, term (≥ 37 weeks), vertex vaginal deliveries and pre-pregnancy BMI ≥ 25.0 kg/m^2^. Women were categorized by GWG based on Institute of Medicine guidelines: below (Group A), within (Group B), or above recommendations (Group C). Exclusion criteria included cesarean delivery, diabetes, multiple gestations, stillbirth, preterm birth, non-vertex presentation, and maternal age < 18 or > 45 years. Perineal injury was defined as any perineal laceration or episiotomy. Univariate and multivariable logistic regression analyses were performed.

**Results:**

Among 5,082 deliveries, 13.1% were in Group A, 28.2% in Group B, and 58.7% in Group C. Episiotomy rates were higher in Group C versus Groups A/B (38.7% vs. 34.7%/34.5%; *p* = 0.011), while overall perineal injury (87.2%–88.5%, *p* = 0.652) and OASI rates (0.5–1.0%, *p* = 0.428) did not differ. In multivariate analysis, GWG was not associated with perineal injury. Independent risk factors included epidural analgesia (OR = 1.39, 95% CI 1.10–1.75), vacuum-assisted delivery (VAD) (OR = 2.83, 95% CI 2.07–3.86), higher birthweight (OR = 1.06 per 100 gr., 95% CI 1.03–1.08), and advanced gestational age (OR = 1.13, 95% CI 1.04–1.23).

**Conclusion:**

GWG was not associated with perineal injury, whereas VAD, epidural analgesia, increased birthweight, and advanced gestational age were independent risk factors.

## What does this study add to the clinical work

**Table:** 

Gestational weight gain does not appear to be a determinant of perineal injury among overweight and obese primiparous women. Instead, intrapartum and fetal characteristics, specifically epidural analgesia, vacuum-assisted delivery, increased birthweight, and advanced gestational age, play a more influential role in predicting perineal injury. Clinicians should prioritize optimizing labor management and perineal protection strategies to mitigate these risks, rather than focusing on GWG as a modifiable factor.	

## Introduction

Perineal injury is a common consequence of vaginal delivery, affecting up to 90% of women postpartum [1, 2]. These injuries are associated with prolonged recovery, including persistent pain, dyspareunia, and limitations in daily functioning [3]. Additionally, perineal injury may increase the risk of local infection and impaired wound healing, particularly among individuals with comorbidities such as obesity [3].

Over the past two decades, the prevalence of obesity among women of reproductive age in the United States has increased substantially, now representing one of the most common health concerns in this population [4]. Multiple studies have specifically examined the relationship between maternal body mass index (BMI), and perineal injury with conflicting results [5–10], whereas the association between GWG and perineal injury remains less clearly defined [10–12].

In 2009, the Institute of Medicine (IOM) provided guidelines for appropriate maternal weight gain based on pre-pregnancy BMI to optimize both maternal and fetal health outcomes [13]. However, the specific association between GWG and perineal injury, particularly among overweight and obese women (BMI ≥ 25.0 kg/m^2^) is not fully understood.

Comprehensive evidence on how GWG, stratified by IOM thresholds, affects overweight and obese women remains limited particularly in large, contemporary cohorts, leaving important questions about optimal weight gain recommendations inadequately addressed.

Thus, we aimed to determine whether GWG categorized according to IOM recommendations, is associated with the incidence and severity of perineal injury, including OASI, in overweight and obese primiparous women, and to identify obstetric and intrapartum factors that may modify this association.

## Methods

### Study design and participants

We conducted a retrospective cohort study at a university-affiliated tertiary medical center. Eligibility was limited to primiparous women with singleton, vertex-presenting pregnancies who delivered between January 2012 and December 2023. Women with a body mass index (BMI) ≥ 25.0 kg/m^2^, recorded either pre-pregnancy or during the initial prenatal visit (up to 12 weeks’ gestation), were eligible for inclusion.

Participants were categorized into three groups based on gestational weight gain (GWG) in accordance with guidelines from the Institute of Medicine (IOM) [13] and the American College of Obstetricians and Gynecologists (ACOG) [14] :Group A – Below recommendations: GWG < 7 kg for overweight women (BMI 25.0–29.9 kg/m^2^), and < 5 kg for women with obesity (BMI ≥ 30.0 kg/m^2^), Group B – Within recommended range: GWG 7–11 kg for overweight women, and 5–9 kg for women with obesity and Group C – Above recommendations: GWG > 11 kg for overweight women, and > 9 kg for women with obesity.

Exclusion criteria were maternal age < 18 or > 45 years, pregestational or gestational diabetes, multiple gestations, stillbirth, preterm birth (< 37 weeks), cesarean delivery, and incomplete GWG data.

The Sultan classification criteria was used to classify perineal tears [15]. prolonged second stage in primiparous was defined as ≥ 2 h without epidural and ≥ 3 h with epidural. According to our institutional protocol a medio-lateral or lateral episiotomy is performed selectively according to the clinical judgment of the obstetrician or a certified midwife. Perineal injury was defined as any perineal trauma, spontaneous (labial, vaginal, perineal, or anal sphincter tears) or iatrogenic (episiotomy).

The primary outcome was the incidence of perineal injury, analyzed across the three GWG groups. Secondary variables included potential risk factors for perineal injury.

### Data collection

Data were retrieved from computerized delivery room logbook containing demographic, obstetric, and clinical information, including maternal age, maternal ethnicity, BMI (pre-gestational), smoking, drug use, gestational weight gain, gestational age at delivery, In-Vitro-Fertilization (IVF) labor onset (spontaneous vs. medical induction), epidural analgesia, fetal head presentation (occiput anterior vs. occiput posterior [OP]), second stage duration, prolonged second stage of labor,vacuum-assisted delivery (VAD), episiotomy, degree of perineal tear and newborn birthweight.

### Statistical analysis

Categorical variables are presented as frequencies and percentages. Continuous variables were assessed for normality using the Kolmogorov–Smirnov test, histograms, and Q–Q plots, and are presented as mean ± standard deviation if normally distributed or as median with interquartile range otherwise. Categorical variables were compared across BMI categories (≥ 25 kg/m^2^) and gestational weight gain groups (below, within, or above recommended levels) using the Chi-square test. Continuous and ordinal variables were compared using independent-samples ANOVA or the Kruskal–Wallis test, as appropriate. Multivariable logistic regression was performed to examine the association between weight gain category and perineal injury adjusting for potential confounders including maternal age and pre-pregnancy BMI [16]. All statistical tests were two-sided, and a *p*-value < 0.05 was considered statistically significant. Analyses were conducted using SPSS software (version 29.0.2; IBM Corp., Armonk, NY, USA, 2023).

This retrospective analysis was approved by the Institutional Review Board of Tel Aviv Sourasky Medical Center (Approval No. TLV-0221–25) and conducted in accordance with the principles of the Declaration of Helsinki (2000). The dataset was fully anonymized prior to analysis, rendering individual informed consent unnecessary according to institutional policies.

## Results

**Fig. 1:**
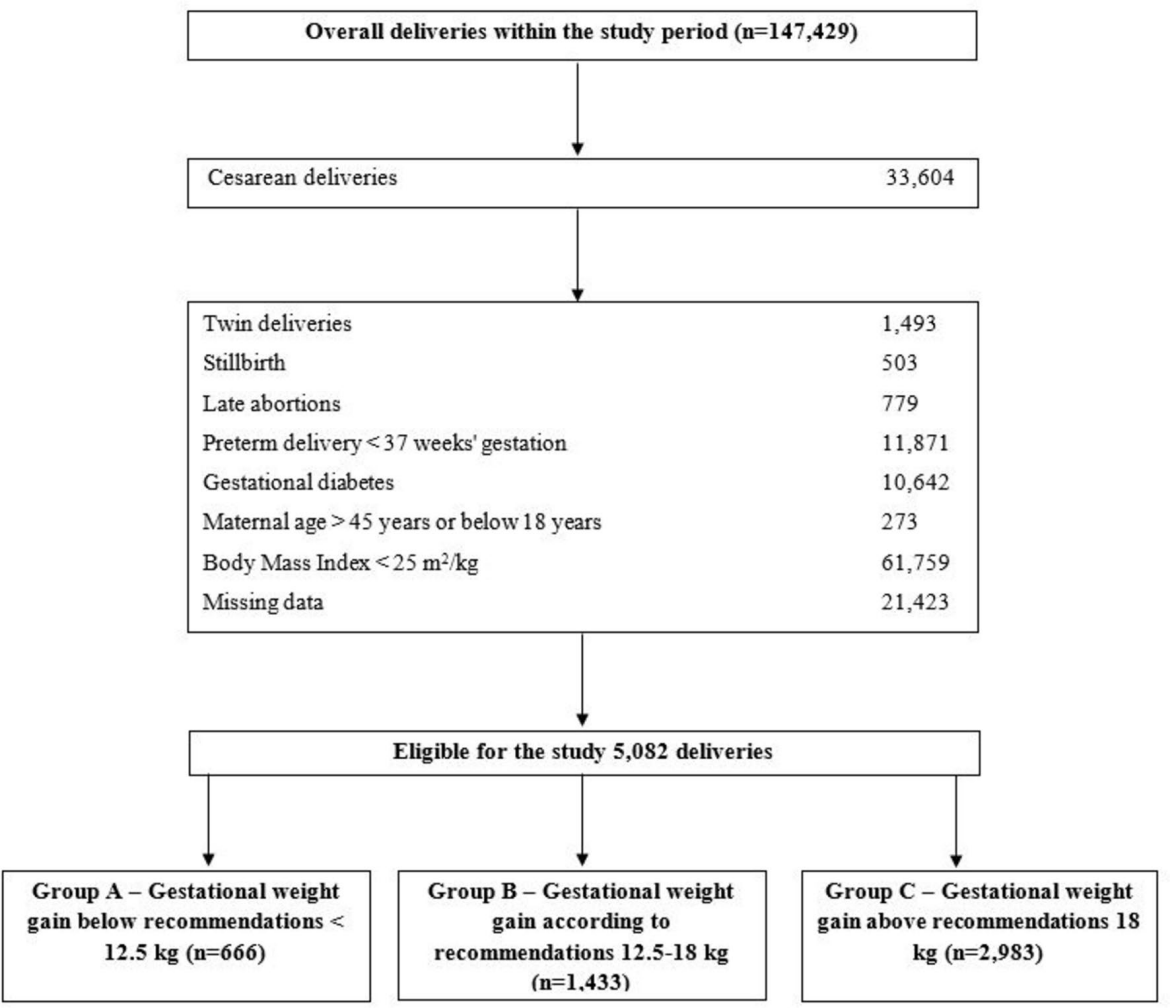
Study design

**Fig. 2:**
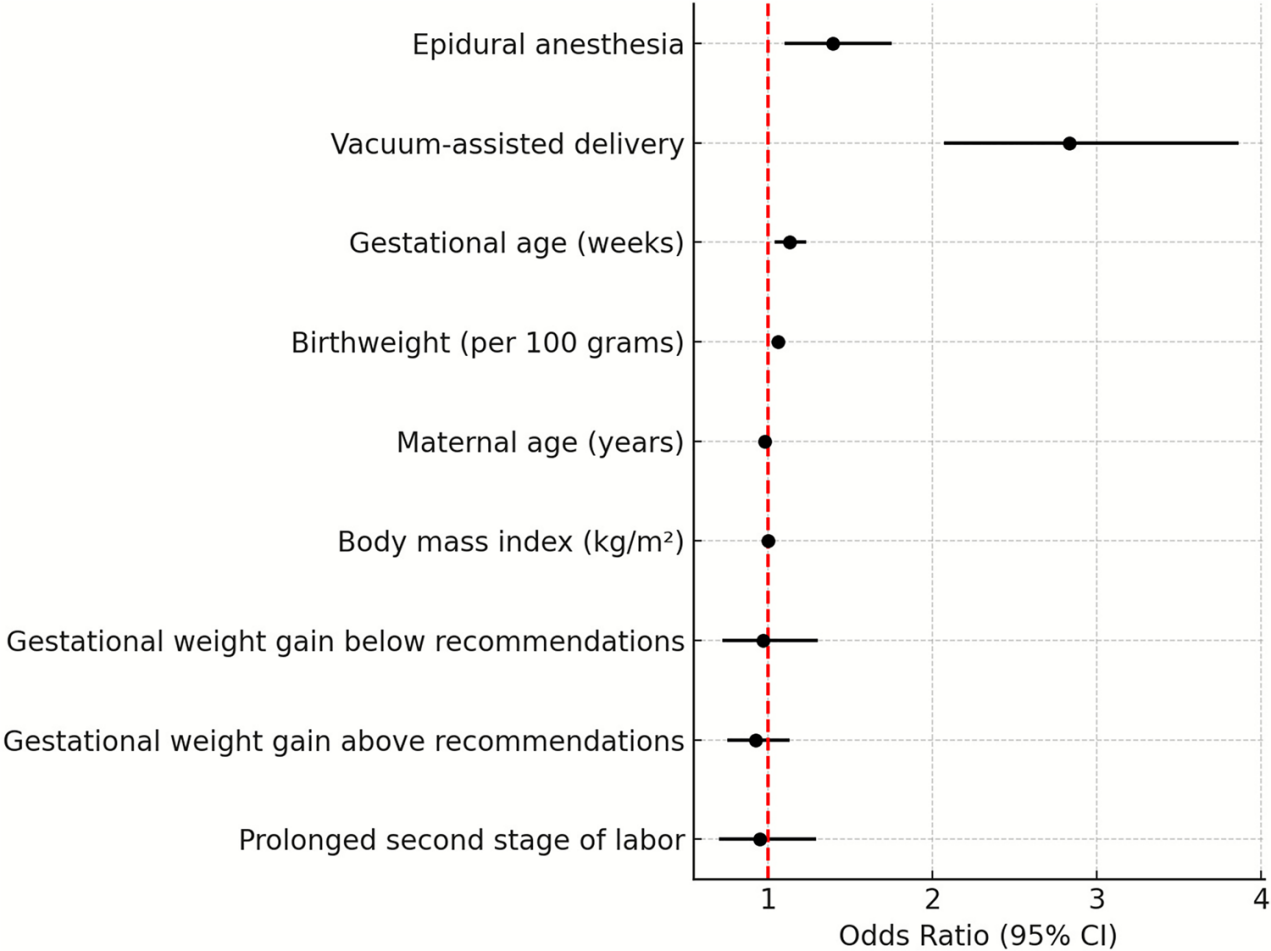
Forest plot— factors associated with perineal injury

During the study period, a total of 147,429 deliveries were recorded, with 5,082 (3.4%) meeting our inclusion criteria. Group A below recommendations comprised 666 women (13.1%), Group B within recommended range included 1,433 women (28.2%), and Group C above recommendations had 2,983 women (58.7%) (Figures 1, 2).

Maternal characteristics are depicted in Table 1. Group A demonstrated a higher mean of BMI (kg/m^2^) compared to groups B & C (29.9 [± 4.6] vs. 28.4 [± 3.4] and 28.0 [± 2.9] respectively, *p* < 0.001).

**Table 1:** Maternal characteristics stratified by gestational weight gain according to institute of medicine guidelines

Characteristic	Group A (*n* = 666)	Group B (*n* = 1,433)	Group C (*n* = 2,983) Mean	*P*-value
Maternal Age (years), (± SD)	30.9 (5.0)	30.8 (4.9)	30.7 (4.5)	0.540
Smoking, *n* (%)	26 (4.1%)	47 (3.4%)	116 (4.0%)	0.565
IVF, *n* (%)	135 (21.2%)	234 (17.0%)	394 (13.8%)	< 0.001
Gestational Age, mean (± SD)	39.8 (1.1)	39.8 (1.1)	40.0 (1.1)	** < 0.001**^**b,c**^
BMI (kg/m^2^), mean (± SD)	29.9 (4.6)	28.4 (3.4)	28.0 (2.9)	** < 0.001**^**a,b,c**^
Maternal Ethnicity				
Caucasian *n* (%)	649 (97.7%)	1400 (97.7%)	2939 (98.5%)	0.139
Asians *n* (%)	9 (1.4%)	17 (1.2%)	30 (1.0%)	
African *n* (%)	6 (0.9%)	16 (1.1%)	14 (0.5%)	
GWG (kg), mean (± SD)	2.5 (3.7)	8.8 (1.6)	16.2 (4.2)	

*SD* = Standard Deviation; *BMI* = body mass index; *IVF* = In-Vitro-Fertilization

When the *p*-value indicates a significant difference between the groups, each superscript letter denotes a subset of two groups of weight gain that differ significantly (*p* < 0.05) from each other: (a) below recommended vs. recommended, (b) below recommended vs. above recommended (c) below recommended vs. above recommended

Significant differences (*P* < 0.05) are presented in BOLD

Table 2 presents labor characteristics. Group C had higher rates of epidural analgesia (87.3% vs. 84.7% and 82.8%, *p* < 0.001) and greater mean birthweight (3342 g ± 389 vs. 3154 g ± 412 and 3241 g ± 398) compared with groups A and B, respectively. In addition, the duration of the second stage of labor was longer in group C (101 ± 64 min) than in groups A and B (92.6 ± 64 and 92.9 ± 64 min, respectively; *p* < 0.001).

**Table 2:** Labor characteristics stratified by gestational weight gain according to institute of medicine guidelines

Characteristic	Group A (*n* = 666)	Group B (*n* = 1,433)	Group C (*n* = 2,983)	*P*-value
Induction of labor, *n* (%)	172 (26.1%)	386 (27.3%)	828 (28.0%)	0.595
Epidural analgesia, *n* (%)	564 (84.7%)	1187 (82.8%)	2605 (87.3%)	** < 0.001**^**c**^
Occiput posterior, *n* (%)	26 (14.2%)	40 (2.8%)	117 (3.9%)	0.152
Second stage duration, mean (± SD)	92.6 (64.6)	92.9 (64.2)	101.0 (64.1)	** < 0.001**^**b,c**^
Prolonged second stage, *n* (%)	70 (10.9%)	172 (12.5%)	389 (13.4%)	0.213
Vacuum assisted delivery, *n* (%)	121 (18.2%)	260 (18.1%)	607 (20.3%)	0.150
Birthweight (g), mean (± SD)	3154 (412)	3241 (398)	3342 (389)	** < 0.001**^**a,b,c**^

*SD* = standard deviation;

When the *p*-value indicates a significant difference between the groups, each superscript letter denotes a subset of two groups of weight gain that differ significantly (*p* < 0.05) from each other: (a) below recommended vs. recommended, (b) below recommended vs. above recommended (c) below recommended vs. above recommended

Significant differences (*P* < 0.05) are presented in BOLD

Table 3 summarizes the rates of perineal injuries, including OASI. Univariate analysis revealed a higher rate of episiotomy in group C compared with groups A and B (38.7% vs. 34.7% and 34.5%, respectively; *p* = 0.011), while no significant differences were observed between groups in the rates of overall perineal injury (87.2–88.5, *p* = 0.652). OASI rates were comparable across groups, ranging from 0.5% to 1.0% (*p* = 0.428).

**Table 3:** Perineal injury rates stratified by gestational weight gain according to institute of medicine guidelines

Characteristic	Group A (*n* = 666)	Group B (*n* = 1,433)	Group C (*n* = 2,983)	*P*-value
Overall perineal injury	581(87.2%)	1263(88.1%)	2640(88.5%)	0.652
Spontaneous perineal lacerations, *n* (%)^a^	415(62.3%)	876(61.1%)	1812(60.7%)	0.693
1st degree, *n* (%)	144 (21.6%)	332 (23.2%)	677 (22.7%)	0.700
2nd degree, *n* (%)	245 (36.8%)	461 (32.2%)	978 (32.8%)	
1st or 2 nd unspecified*, *n* (%)				
3rd degree, *n* (%)	2 (0.3%)	11 (0.8%)	29 (1.0%)	
4th degree, *n* (%)	1 (0.2%)	2 (0.1%)	0 (0%)	
OASI, *n* (%)	3 (0.5%)	13 (0.9%)	29 (1.0%)	0.428
Labial Tear, *n* (%)	62(9.3%)	142(9.9%)	267(9.0%)	0.589
Episiotomy, *n* (%)	231(34.7%)	495(34.5%)	1155(38.7%)	**0.011**^**b,c**^

^*^Unspecified—1st or 2nd degree tears not classified separately, not involving the anal sphincter

When the *p*-value indicates a significant difference between the groups, each superscript letter denotes a subset of two groups of weight gain that differ significantly (*p* < 0.05) from each other: (a) below recommended vs. recommended, (b) below recommended vs. above recommended (c) below recommended vs. above recommended

Significant differences (*P* < 0.05) are presented in BOLD

OASI- Obstetric Anal Sphincter Injury

Multivariate logistic regression analysis (Table 4) revealed that neither insufficient nor excessive GWG was significantly associated with increased risk for perineal injury when compared to normal GWG. Factors associated with increased risk for perineal injury included epidural analgesia (OR = 1.39, 95% CI [1.10–1.75], *p* = 0.006), VAD (OR = 2.83, 95% CI [2.07–3.86], *p* < 0.001), increased birthweight (OR = 1.06 per 100 g. 95% CI [1.03–1.08], *p* < 0.001, and advanced gestational age (OR = 1.13, 95% CI [1.04–1.23], *p* = 0.006).

**Table 4:** Risk factors for perineal injury: multivariate logistic regression in overweight primiparous women

Variable	OR	95% CI	*P*-value
Epidural analgesia	1.39	1.10–1.75	**0.006**
Vacuum assisted delivery	2.83	2.07–3.86	** < 0.001**
Gestational age (weeks)	1.13	1.04–1.23	**0.004**
Birthweight (per 100 g)	1.06	1.03–1.08	** < 0.001**
Age (years)	0.98	0.96–1.0	**0.025**
BMI (kg/m2)	1.0	0.98–1.04	0.497
Normal weight gain (reference)	1.000		
Gestational weight gain below recommendation	0.97	0.72–1.30	0.700
Gestational weight gain above recommendation	0.92	0.75–1.13	
Prolonged Second stage of labor	0.95	0.70–1.29	0.755

Significant differences (*P* < 0.05) are presented in BOLD

## Discussion

### Principal findings

This study aimed to assess the association between GWG and the incidence of perineal injury among overweight and obese primiparous women. The key findings were: (1) neither insufficient nor excessive GWG was associated with increased risk of perineal injury; (2) excessive GWG was associated with a higher rate of episiotomy; and (3) risk factors for perineal injury included epidural analgesia, VAD, higher birthweight, and advanced gestational age.

### Results in the context of what is known

Among pregnant individuals, both elevated pre-pregnancy BMI and GWG are known to increase the risk of adverse maternal and neonatal outcomes [14, 17, 18]. The Institute of Medicine (IOM) guidelines recommend tailoring GWG to pre-pregnancy BMI to optimize perinatal outcomes [13].

The relationship between BMI and perineal injury has been previously explored, with some studies suggesting a protective effect of higher BMI on the risk of perineal tears [5, 7, 19], However, data regarding the impact of GWG on perineal outcomes remain limited. In our cohort, the overall incidence of perineal lacerations ranged from 87.2% to 88.5%, and the rate of obstetric anal sphincter injury (OASI) was 0.9%, aligning with previously reported findings [20, 21]. The relatively low OASI rate in our study may reflect the previously described protective association of increased BMI [5, 19]. Nonetheless, this study did not identify a statistically significant protective or adverse effect of GWG on the risk of perineal injury. Gallagher et al. similarly reported that neither obesity (BMI ≥ 30 kg/m^2^) nor excessive GWG above IOM recommendations was associated with increased incidence or severity of perineal lacerations in nulliparous women. However, their analysis evaluated BMI and GWG categories independently, leaving unresolved whether GWG within an already elevated BMI range modifies perineal injury risk [12].

While some studies reported lower rates of minor perineal injury among women with obesity and no association between BMI and OASI [5], others highlighted the role of prolonged second stage and epidural analgesia as significant risk factors for perineal lacerations [22]. Our findings are consistent with the latter, identifying epidural use and fetal factors, including birthweight and gestational age, as key contributors to perineal injury.

Notably, although GWG by itself was not significantly associated with increased risk of perineal injury, it was linked to higher rates of episiotomy. This finding may be explained in part by the higher birthweights and more frequent use of epidural analgesia observed among women who exceeded recommended GWG, both established risk factors for episiotomy. Despite slightly longer durations of the second stage of labor in this group (mean difference < 10 min), excessive GWG was not significantly associated with a prolonged second stage or an increased likelihood of VAD. These results suggest that the association between GWG and episiotomy may be mediated by fetal and labor-related characteristics rather than GWG itself.

Other studies emphasized that risk factors for second-degree perineal tears and OASI include nulliparity, fetal macrosomia, advanced gestational age, and operative vaginal birth [2, 5, 8, 10, 22]. Our findings mostly align with these established associations. Although maternal age demonstrated a statistically significant association with perineal injury (OR = 0.98, *p* = 0.025), the effect size was very small. Therefore, this finding is unlikely to hold clinical relevance and should be interpreted with caution. Nonetheless, it is noteworthy that in our cohort, advanced maternal age does not appear to be a risk factor for perineal trauma in this population.

The role of epidural analgesia remains debated in the literature [1, 23, 24]. However, in our cohort it emerged as an independent risk factor for perineal injury among overweight women. Epidural analgesia may influence perineal outcomes through several physiological and mechanical pathways. Pain relief can reduce maternal urge to push and modify motor control during the second stage, leading to a prolonged expulsive phase and an increased likelihood of perineal tears. Relaxation of pelvic floor tone under regional anesthesia may also predispose the tissues to laceration [24].

While our study demonstrated relatively high rates of episiotomy, existing data report 40–50% for operative vaginal births [25], and as high as 89% in some studies [26], which ultimately aligns with our findings.

### Clinical Implications

This study suggests that gestational weight gain (GWG), whether below, within, or above the Institute of Medicine (IOM) recommendations, does not appear to be independently associated with a significantly increased risk of perineal injury among overweight and obese primiparous women. However, the findings indicate that intrapartum factors, particularly epidural analgesia, VAD, higher birthweight, and advanced gestational age—may play a more prominent role in influencing perineal injury. These results highlight the importance of careful intrapartum management and individualized assessment of risk, rather than focusing solely on maternal weight gain. Preventive strategies, such as optimized perineal support and prudent use of operative interventions, may help reduce the burden of perineal morbidity in this population. Although GWG remains an important component of overall maternal and fetal health, its role as a direct predictor of perineal injury risk appears limited based on the current evidence.

### Research Implications

Further studies are needed to explore the relationship between maternal obesity class and perineal injury risk, as the current analysis did not stratify outcomes by obesity severity. Prospective multicenter research including more ethnically and demographically diverse populations would enhance the generalizability of these findings. Incorporating objective measures such as pelvic floor ultrasound or biomechanical tissue assessment may help elucidate the mechanisms linking maternal body composition, soft tissue elasticity, and perineal outcomes. Future work should also investigate how institutional labor management practices, particularly episiotomy policies and perineal support techniques, influence perineal injury rates across different BMI and GWG categories.

### Strengths and limitations

This study possesses several strengths that enhance the validity and relevance of its findings. It includes a large, well-characterized cohort of primiparous women over an extended period, enabling robust statistical analysis. Detailed clinical data were obtained from standardized delivery records, providing accurate information on maternal BMI and other key variables. By focusing specifically on overweight and obese individuals, the study offers valuable insights into a population at increased risk of perineal injury. The application of consistent institutional protocols for labor assessment and delivery management enhances internal validity by reducing variability in clinical care. Furthermore, the exclusion of women with pregestational or gestational diabetes was a deliberate effort to isolate the specific impact of GWG on perineal outcomes, minimizing confounding by metabolic comorbidities.

However, the study also has limitations. The retrospective design inherently relies on the accuracy and completeness of recorded data, which may affect data reliability. The single-center setting, predominantly serving a homogeneous Caucasian population, limits the generalizability of the findings to more diverse populations. In addition, although the study targeted overweight and obese individuals, it did not stratify outcomes by obesity class, which may have masked potential differences in risk profiles across BMI subgroups. Future studies should consider differentiating between obesity classes to explore potential dose–response relationships. Lastly, the observational nature of the study precludes causal inference, and the possibility of residual confounding cannot be excluded.

To our knowledge, this is the first study to evaluate the association between GWG—categorized according to IOM and ACOG guidelines—and perineal injury among overweight and obese primiparous individuals. Despite its limitations, the findings offer clinically relevant insights that may inform counseling, risk assessment, and guide future prospective investigations.

## Conclusion

This study highlights that among overweight women, gestational weight gain did not show a direct correlation with perineal injury, while other factors such as epidural analgesia, vacuum-assisted deliveries, increased birthweight, and advanced gestational age were associated with a higher risk of perineal injury.

## Data Availability

The datasets generated and/or analyzed during the current study are available from the corresponding author upon reasonable request.
